# The Impact of Lipoprotein-Associated Oxidative Stress on Cell-Specific Microvesicle Release in Patients with Familial Hypercholesterolemia

**DOI:** 10.1155/2016/2492858

**Published:** 2016-01-27

**Authors:** M. H. Nielsen, H. Irvine, S. Vedel, B. Raungaard, H. Beck-Nielsen, A. Handberg

**Affiliations:** ^1^Danish PhD School of Molecular Metabolism, University of Southern Denmark, 5000 Odense, Denmark; ^2^Department of Clinical Biochemistry, Aalborg University Hospital, 9000 Aalborg, Denmark; ^3^Department of Medicine and Cardiology A, Aarhus University Hospital, 8000 Aarhus, Denmark; ^4^Department of Radiology, Aarhus University Hospital, 8000 Aarhus, Denmark; ^5^Department of Cardiology, Aalborg University Hospital, 9000 Aalborg, Denmark; ^6^Department of Endocrinology M, University of Southern Denmark, 5000 Odense, Denmark; ^7^Department of Clinical Medicine, Faculty of Medicine, Aalborg University, 9000 Aalborg, Denmark

## Abstract

*Objective*. Microvesicles (MVs) are small cell-derived particles shed upon activation. Familial hypercholesterolemia (FH) particularly when associated with Achilles tendon xanthomas (ATX) predisposes to atherosclerosis, possibly through oxLDL-C interaction with the CD36 receptor. To investigate the hypothesis that MVs derived from cells involved in atherosclerosis are increased in FH and that CD36 expressing MVs (CD36+ MVs) may be markers of oxLDL-C-induced cell activation, cell-specific MVs were measured in FH patients with and without ATX and their association with atherogenic lipid profile was studied.* Approach and Results*. Thirty FH patients with and without ATX and twenty-three controls were included. Plasma concentrations of MVs and CD36+ MVs derived from platelets (PMVs), erythrocytes (ErytMVs), monocytes (MMVs), and endothelial cells (EMVs), as well as tissue factor-positive cells (TF+ MVs), were measured by flow cytometry. Total MVs, MMVs, EMVs, ErytMVs, and TF+ MVs were significantly increased in FH patients, compared to controls. CD36+ MVs derived from endothelial cells and monocytes were significantly higher in FH patients and oxLDL-C predicted all the investigated cell-specific CD36+ MVs in FH patients with ATX.* Conclusions*. MVs derived from cells involved in atherosclerosis were increased in FH and may contribute to elevated atherothrombosis risk. The increased cell-specific CD36+ MVs observed in FH may represent markers of oxLDL-C-induced cell activation.

## 1. Introduction

Familial hypercholesterolemia (FH) is an autosomal dominant genetic disorder associated with elevated levels of low-density lipoprotein-cholesterol (LDL-C), cholesterol deposits in tendons (xanthomas), and premature heart disease [1, 2]. The presence of tendon xanthomas (TX) is a marker of increased cardiovascular disease risk among patients with FH [3, 4]. Although the underlying mechanisms are not entirely understood, proatherogenic modifications of lipoproteins such as oxidized LDL-cholesterol (oxLDL-C) have been attributed to important roles in both TX development [5, 6] and scavenger receptor CD36-mediated atherosclerosis [7–10]. The activating/cytotoxic effect of oxLDL-C has previously been described [11, 12] and more recent studies demonstrate a relationship between proatherogenic factors, including oxLDL-C, and the shedding of submicron membrane vesicles [13–16]. These cell-specific microvesicles (MVs) are shed from the membrane of activated circulating and vascular cells [17, 18] and have in recent years received attention due to their potential involvement in inflammatory and autoimmune diseases, as well as in cardiovascular disorders and the metabolic syndrome [19–22]. They express markers from their parental cell and harbor membrane and cytoplasmic proteins as well as bioactive lipids implicated in a variety of cellular mechanisms [23]. Circulating MVs may therefore be looked upon as biomarkers, providing information about their cellular origin as well as the condition of the parental cell, and as active players in disease development and progression [24–26]. CD36 is expressed on various types of cells involved in processes leading to atherosclerosis.

We hypothesize that circulating MVs derived from cells involved in atherosclerosis development are increased with elevated atherosclerosis risk in FH. In particular, we propose that MVs expressing CD36 may be markers of oxLDL-C-induced cell activation potentially leading to cholesterol accumulation in the vessel wall. To explore this hypothesis, circulating cell-specific MVs were measured in hypercholesterolemic FH patients with and without Achilles tendon xanthomas (ATX). Furthermore, the potential association between oxLDL-C and the presence of cell-specific CD36-positive MVs was studied.

## 2. Materials and Methods

### 2.1. Study Subjects

The study group comprising thirty patients (18 females and 12 males) genetically diagnosed with heterozygous FH and twenty-three healthy controls (15 females and 8 males) as indicated by a medical questionnaire has previously been described [6]. FH patients were selected on the presence (11 females and 5 males) or absence (7 females and 7 males) of ATX, according to medical records. None of the participants were taking medication at inclusion, except lipid-lowering therapy, which was withdrawn 8 weeks before study start. The study was conducted in agreement with the Helsinki II declaration and approved by the Central Denmark Region Committees on Health Research (2010-0147) and by the Danish Data Protection Agency (j. number 2010-41-4879). The presence of ATX was confirmed by ultrasonographic measurements as described in [6].

Blood pressure, height, and body weight were recorded and blood samples were obtained in the fasting state. The following parameters were determined in the routine laboratory: platelet, leukocyte, and monocyte counts, as well as hemoglobin levels were determined on a Sysmex XE-5000*™* Automated Hematology System in ethylenediaminetetraacetate (EDTA) blood. A Cobas® 6000 analyzer (Roche) was used for analysis of glucose, alanine-aminotransferase (ALT), triglycerides, total cholesterol (Total-C), low-density lipoprotein-cholesterol (LDL-C), high-density lipoprotein-cholesterol (HDL-C), and apolipoprotein B (ApoB) in lithium-heparin plasma. LDL-C concentrations were estimated from the Friedewald formula [27].

### 2.2. Determination of oxLDL-C Levels

EDTA plasma for measurement of oxLDL-C was stored at −80°C until being assayed. The Mercodia ELISA assay (Mercodia, Uppsala, Sweden) used for oxLDL-C analysis measures both minimally and fully oxidized LDL-C particles. The intra- and interassay coefficients of variation were 5.5–7.3% and 4.0–6.2%, respectively, according to the manufacturer.

### 2.3. Flow Cytometric Measurement of Analysis Microvesicles in Plasma

MV subpopulations were detected and quantitated by a previously reported method [28]. Blood samples for MV preparation were collected into sodium citrate anticoagulant at a 3.2% (0.105 M) final concentration and processed within 1 hour. Platelet-free plasma (PFP) was prepared by serial centrifugations (10 min. at 1800 ×g, 15 min. at 3000 ×g, and 5 min. at 3000 ×g), frozen, and stored at −80°C until analysis. For each analysis, 50 *μ*L of freshly thawed PFP was transferred to a TruCount*™* tube (BD Biosciences, New Jersey, USA) containing a lyophilized pellet, which releases a known number of fluorescent beads, used for MV quantification following the manufacturer's instructions. Subsequently, MVs were labeled by adding 10 *μ*L fluorescein isothiocyanate- (FITC-) conjugated Lactadherin (83 *μ*g mL^−1^, Haematologic Technologies Inc., Vermont, USA) [29, 30], characterized by a phosphatidylserine- (PS-) bonding motif [29, 30].

To identify the cellular origin of MVs the following fluorescent mAbs to specific cell surface markers were added immediately after Lactadherin-FITC labeling: 8 *μ*L Allophycocyanin- (APC-) conjugated anti-human CD41 (6 *μ*g mL^−1^ IgG1, *κ* (clone HIP8, BioLegend, San Diego, CA, USA)) was used to detect platelet-derived MVs (PMVs), 5 *μ*L anti-human CD235a-APC (3 *μ*g mL^−1^ IgG2b, *κ* (clone HIR2, eBioscience, San Diego, CA, USA)) was used to detect erythrocyte-derived MVs (ErytMVs), 8 *μ*L APC-conjugated anti-human CD31 (50 *μ*g mL^−1^ IgG1, *κ* (clone WM59, BioLegend)) and 3 *μ*L PerCP-conjugated anti-human CD42b (400 *μ*g mL^−1^ IgG1, *κ* (clone HIP1, BioLegend)) were used to detect endothelial cell-derived MVs (EMVs) which were defined as particles positively labeled by CD31 mAbs (platelet and endothelium surface antigen) and negative for CD42b (platelet surface antigen) [31]. 5 *μ*L Phycoerythrin- (PE-) conjugated anti-human CD36 (6.25 *μ*g mL^−1^ IgG2a, *κ* (clone 5–271)) was used to detect CD36-positive MVs (CD36+ MVs) and 20 *μ*L Phycoerythrin- (PE-) conjugated anti-human CD142 (12.5 *μ*g mL^−1^ IgG1, *κ* (clone HTF-1, BD Pharmingen, New Jersey, USA)) was used to detect tissue factor-positive MVs (TF+ MVs).

After 30 min. of incubation (4°C, in the dark), 250 *μ*L 0.22 *μ*m filtered PBS was added to each labeled sample. Plasma samples were analyzed immediately after labeling using a BD FACSAria*™* III High Speed Cell Sorter, which incorporates three air-cooled lasers at 488, 633, and 407 nm wavelengths, and equipped with BD FACSDiva*™* software (v. 6.1.3). Logarithmic amplification was used for all channels and isotype controls on plasma samples were used as negative controls. For the detection of MVs an initial microvesicle-size gate was established by pilot standardization experiments using a blend of size-calibrated fluorescent beads, with sizes ranging from 0.2-*μ*m (Invitrogen, Hellerup, Denmark) to 3.0-*μ*m (Megamix beads (0.5, 0.9, and 3.0-*μ*m), Biocytex, Marseille, France). The upper limit of the MV gate was set just above the size distribution of the 0.9-*μ*m beads in an forward (FSC-A) and side scatter (SSC-A) setting (log scale) using the “autogate” function inside the FlowJo*™* (v. 8.8.7, Tree Star, Inc., Oregon, USA) software. The lower limit was the noise threshold of the instrument. This threshold was set at the SSC-A parameter to prevent exclusion of the smallest events. We defined MVs as particles that were less than 1.0 *μ*m in diameter, had positive staining for Lactadherin-FITC, and expressed cell-specific markers. Increased MMV number has previously been reported on the same FH study population [6].

### 2.4. Statistics

Statistical analyses were carried out using the STATA 11.2 statistical program (StataCorp LP, Texas, USA). MV data were expressed as median (interquartile range) and analyzed using nonparametric Mann-Whitney *U* tests. Continuous variables with a normal distribution were expressed as the mean ± standard deviation and analyzed with Student's *t*-test. Statistical correlation was analyzed using Spearman's rank correlation for nonnormal distributed data. Multiple regression (robust) analysis was used to adjust for the confounding effect of gender. The standardized regression coefficients beta (*β*) is included as a measure of how strongly each predictor variable influences the criterion (dependent) variable. Variables with nonnormal distribution were log-transformed to achieve normal distribution before regression analysis. Shapiro-Wilk's *W* test was used to test the assumption of normality. *p* values are two-sided and considered significant when <0.05.

## 3. Results

### 3.1. Characteristics of the Study Subjects

Table 1 shows the clinical characteristics of the study participants. Total cholesterol, LDL-C, and oxLDL-C were significantly increased in FH patients compared to healthy controls, and HDL-C levels were significantly lower. FH patients with ATX had significantly higher concentrations of total cholesterol, LDL-C, and oxLDL-C than FH patients without ATX, whereas HDL-C levels were similar. All other laboratory measurements were within age- and gender-specific reference intervals, and no significant differences were observed between groups.

### 3.2. Analysis of MV Subpopulations in Patients with FH

**Figure 1:**
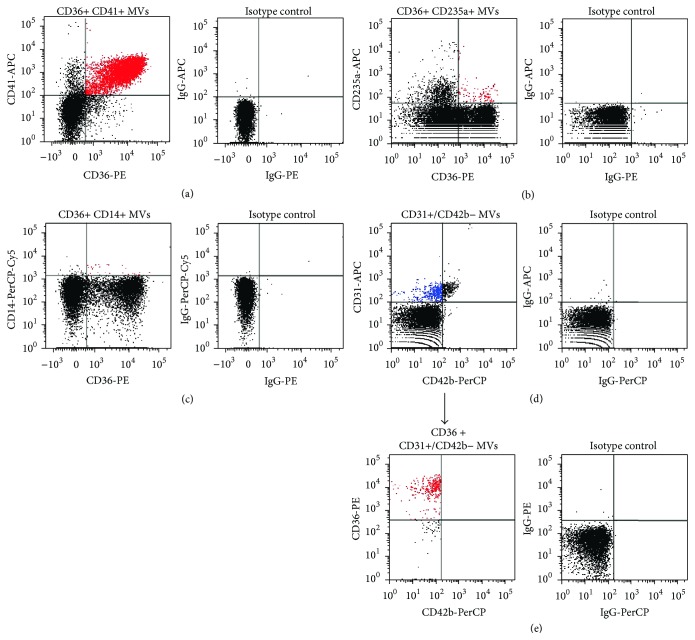
Flow cytometric analysis of CD36-positive MVs derived from various cell types. MVs were identified by size (<1 *μ*m) and by surface exposure of phosphatidylserine. Colabeling with cell-specific markers identified CD36 expressing MVs (colored red) from various cell types, including (a) CD36+ CD41+ (platelet-derived) MVs, (b) CD36+ CD235a+ (erythrocyte-derived) MVs, (c) CD36+ CD14+ (monocyte-derived) MVs, and (d-e) CD36+ CD31+/CD42b− (endothelial cell-derived) MVs identified by a two-color method. CD31 is expressed on both platelets and endothelial cells, whereas CD42b is restricted to platelets, allowing discrimination between PMVs and EMVs (colored blue). Right columns correspond to the respective isotype controls.

FH patients had higher concentrations of total MVs (*p* = 0.022), endothelial cell-derived MVs (EMVs) (*p* = 0.002), erythrocyte-derived MVs (ErytMVs) (*p* = 0.043), monocyte-derived MVs (MMVs) (*p* = 0.004), and tissue factor-positive MVs (TF+ MVs) (*p* = 0.006), when compared to healthy controls (Table 2). No significant difference in platelet-derived MVs (PMVs) was observed. As shown in Figure 1, CD36+ MVs were derived from all the investigated cells and most abundantly among MVs derived from platelets, endothelial cells, and monocytes (Table 2). FH patients had higher concentrations of CD36+ EMVs (*p* = 0.008) and CD36+ MMVs (*p* = 0.001), whereas no significant difference in CD36+ PMVs, CD36+ ErytMVs, or total CD36+ MVs was observed. The presence of ATX had no influence on MV levels (data not shown).

### 3.3. Association between oxLDL-C and CD36-Positive MVs in FH Patients with ATX

OxLDL-C was not correlated with any of the investigated CD36+ MV subpopulations in healthy individuals or in FH patients without ATX. In FH patients with ATX, oxLDL-C showed strong correlations with total CD36+ MVs (Rho = 0.70, *p* = 0.003), CD36+ EMVs (Rho = 0.77, *p* < 0.001), CD36+ PMVs (Rho = 0.74, *p* = 0.001), and CD36+ ErytMVs (Rho = 0.74, *p* = 0.001), but not with CD36+ MMVs. No correlation between oxLDL-C and CD36-negative subpopulations was observed in any of the three study groups.

Gender-adjusted regression analysis demonstrated strong associations between oxLDL-C and all the investigated CD36-positive MVs, including total CD36+ MVs (*R* ^2^ = 0.76, *p* < 0.001), CD36+ EMVs (*R* ^2^ = 0.68, *p* < 0.001), CD36+ MMVs (*R* ^2^ = 0.41, *p* = 0.027), CD36+ PMVs (*R* ^2^ = 0.64, *p* < 0.001), and CD36+ ErytMVs (*R* ^2^ = 0.51, *p* = 0.003) in FH patients with ATX (Table 3). The association with oxLDL-C remained significant for both CD36+ PMVs (*R* ^2^ = 0.70, *p* = 0.004) and CD36+ MMVs (*R* ^2^ = 0.41, *p* = 0.034) after adjusting for platelet and monocyte count, respectively. TF+ MVs and oxLDL-C were significantly associated (*R* ^2^ = 0.26, *p* = 0.031) in FH patients with ATX.

## 4. Discussion

The interest in cell-derived MVs and their potential role in cardiovascular disease (CVD) is steadily increasing, as they may not only reflect the presence of CVD but also play a causative role in its development [32, 33]. This study shows that FH patients have significantly higher concentrations of overall circulating MVs and in particular higher concentrations of those derived from endothelial cells, monocytes, and erythrocytes. Moreover, CD36+ MVs derived from endothelial cells and monocytes were significantly higher in FH patients compared to healthy controls. This study also shows that among FH patients with ATX oxLDL-C was a significant predictor of all the investigated subpopulations of CD36+ MV, whereas MVs negative for CD36 were unrelated to oxLDL-C levels. To the best of our knowledge this is the first study investigating the impact of FH on level and distribution of circulating MVs, in particular CD36+ MVs, and directly addressing the relationship between plasma levels of proatherogenic oxLDL-C and circulating CD36+ MV subpopulations.

Elevated LDL-C is a well-known risk factor for atherosclerosis and CVD [34]. Prolonged lipid exposure to cells within the vascular compartments may trigger the release of MVs in concordance with our observation of increased MV concentrations in FH patients.

This is supported by a recent study demonstrating reduced MV concentrations in FH patients after an effective lipid-lowering treatment with statins [35]. When comparing FH patients to non-FH hypercholesterolemic patients MV concentration was higher in the former indicating that both degree and length of exposure affect MV release [36]. Together, these results indicate that overall MVs have the potential of being a marker of hyperlipidemic atherosclerosis risk.

Cell-specific MVs may have some value as markers for the ongoing processes within the vascular compartment, as reported in [23, 37–39]. The finding herein of increased EMV concentrations in FH patients may therefore indicate endothelial cell activation and dysfunction. The increased MMV concentrations most likely reflect activation of inflammatory cells, whereas increased ErytMV concentrations may be associated with prothrombotic risk. Although our study cannot prove causality because of its cross-sectional nature the results presented herein may provide information about the steady-state condition in the vascular compartment during prolonged exposure to elevated LDL-C levels.

Monocyte-derived MVs are like endothelial-derived and erythrocyte-derived MVs significant sources of procoagulant activity due to their expression of tissue factor [40, 41], as well as of phosphatidylserine, a cofactor in the coagulation cascade [42]. Elevated levels of monocyte-derived TF+ MVs observed in hyperlipidemia patients are suggested to contribute to arterial thrombosis after rupture of atherosclerotic plaques [42]. Moreover, Wang and colleagues recently demonstrated that monocyte-derived MVs could activate endothelial cells in an IL-1 beta-dependent manner [43]. The observed higher concentrations of both MMVs and overall TF+ MVs in FH patients may promote or even accelerate atherothrombosis as suggested in [44].

The association between TF+ MMVs and oxLDL-C has previously been demonstrated in acute coronary syndrome [13]. Moreover, a recent study by Owens et al. reported oxLDL-C-induced TF expression in THP-1 monocytic cells and human monocytes and showed that FH patients had elevated levels of both oxLDL-C and TF+ MV-associated procoagulant activity [45].

The relationship between serum cholesterol and circulating erythrocytes recently reported in 4,469 adult participants of the National Health and Nutrition Examination Survey (NHANES) [46] suggested that erythrocyte viability is related to cholesterol levels. Erythrocyte membranes contain a ratio of cholesterol and phospholipid, which affects their deformability, and without a nucleus and organelles to synthesize lipids, a large proportion of membrane cholesterol content originates from plasma lipids [47]. Changes in shape and deformability may lead to the production of ErytMVs and increased concentrations in FH patients, as presented herein. A possible consequence may be activation of the vascular endothelium, resulting in vascular inflammation and atherosclerosis as previously suggested [48].

Proatherogenic modifications of lipoproteins such as the oxidation of LDL-C are attributed to important roles in the atherogenic process. The activating effect of oxLDL-C causing membrane vesiculation has been described in endothelial cells [49], platelets [14], and monocytes [13, 50], and in a previous published work from our group we reported that high numbers of MMVs are associated with proinflammatory monocytes and oxLDL-C [6]. The binding of oxLDL-C to CD36 is considered to trigger intracellular signaling events, but the precise contribution of CD36 is still a matter of debate [51, 52]. Knowing that CD36 expression is a common feature of these cell types, we propose that oxLDL-C binding to CD36 may lead to cell activation and MV release. The increased MV concentrations found in FH patients and the strong association between CD36+ MVs and oxLDL-C in FH with ATX suggest a possible involvement in processes leading to endothelial dysfunction and cardiovascular risk.

This study has some limitations as we used 900 nm polystyrene microspheres from Megamix to define the upper limit of a forward scatter MV size gate. Although this gating strategy has been published by the ISTH Scientific Standardization Committee [53, 54] the difference in refractive index and thus forward angle light scatter between polystyrene beads and cellular MVs must be taken into account, as stated in a very recent study by van der Pol et al. [55].

In conclusion, increased circulating MVs, in particular MMVs, EMVs, ErytMVs, and TF+ MVs, may contribute to atherosclerosis development and elevated atherothrombosis risk in patients with FH. The increased endothelial- and monocyte-derived CD36+ MVs may reflect the state of their parental cells and indicate oxLDL-C-induced activation through a CD36-dependent mechanism. The association between oxLDL-C and CD36+ MVs in FH patients with ATX suggests the involvement of lipoprotein-associated oxidative stress in MV release. Thus, although the prognostic potential of circulating MVs is still in its infancy, our findings indicate that detection and quantification of MV subpopulations, especially CD36+ MVs, may be a potentially valuable tool for risk assessment and may add to a better understanding of their potential contribution to vascular complications in hypercholesterolemia.

## Figures and Tables

**Table 1:** Characteristics of study participants.

Characteristics	Controls	FH	*p* value^a^	FH ATX−	FH ATX+	*p* value^b^
	(*n* = 23)	(*n* = 30)		(*n* = 14)	(*n* = 16)	
Men/women	8/15	12/18	NS	7/7	5/11	NS
Age (years)	47.0 ± 10.1	45.5 ± 9.1	NS	43.1 ± 10.2	47.5 ± 7.8	NS
BMI (kg/m^2^)	23.7 ± 3.3	25.5 ± 5.2	NS	25.1 ± 4.2	25.9 ± 5.9	NS
Systolic b.p. (mm Hg)	120.2 ± 10.8	124.9 ± 14.3	NS	122.4 ± 13.7	127.1 ± 14.8	NS
Diastolic b.p. (mm Hg)	74.6 ± 6.7	77.4 ± 6.5	NS	76.0 ± 5.5	78.7 ± 7.1	NS
Platelet count (10^9^/L)	239.8 ± 50.7	248.6 ± 60.1	NS	247 ± 55.7	250 ± 65.4	NS
Leukocyte count (10^9^/L)	5.1 ± 1.2	5.4 ± 1.9	NS	5.0 ± 1.6	5.7 ± 2.2	NS
Monocyte count (10^9^/L)	0.4 ± 0.1	0.5 ± 0.1	NS	0.4 ± 0.1	0.5 ± 0.2	NS
Hemoglobin (mmol/L)	8.7 ± 0.6	9.0 ± 0.8	NS	9.1 ± 1.0	8.9 ± 0.7	NS
Total-C (mmol/L)	5.1 ± 0.7	9.1 ± 1.8	<0.0001	8.2 ± 1.1	9.9 ± 2.0	0.0098
LDL-C (mmol/L)	2.9 ± 0.5	7.0 ± 1.8	<0.0001	6.0 ± 1.1	7.9 ± 1.8	0.0025
HDL-C (mmol/L)	1.7 ± 0.4	1.5 ± 0.4	0.014	1.5 ± 0.5	1.4 ± 0.4	NS
oxLDL-C (U/L)	50.0 ± 11.8	100.3 ± 24.2	<0.0001	83.7 ± 13.6	114.8 ± 22.1	0.0001
ApoB (g/L)	0.8 ± 0.1	1.7 ± 0.4	<0.0001	1.4 ± 0.3	1.8 ± 0.3	0.0015
Glucose (mmol/L)	5.4 ± 0.5	5.5 ± 0.4	NS	5.5 ± 0.4	5.5 ± 0.4	NS
ALT (U/L)	19.0 (14.0–21.0)	19.0 (17.0–34.0)	NS	20.5 (18–32)	18.5 (15.0–36.5)	NS
TG (mmol/L)	1.0 (0.8–1.2)	1.1 (0.8–1.8)	NS	1.3 (0.7–1.8)	1.1 (1.0–1.6)	NS

Normal and nonnormal distributions are shown as the mean ± standard deviation and as the median (interquartile range), respectively. ^a^Controls versus FH subjects; ^b^FH subjects without ATX versus FH subjects with ATX. BMI, body mass index; ALT, alanine-aminotransferase, TG, triglycerides; ApoB, apolipoprotein B; Total-C, total cholesterol; LDL-C, LDL-cholesterol; HDL-C, HDL-cholesterol; oxLDL-C, oxidized LDL-C; b.p., blood pressure; NS, nonsignificant.

**Table 2:** Plasma levels of circulating MVs.

	Controls (*n* = 23)	FH (*n* = 30)	*p* value
Total MVs	15603 (10549–45211)	35616 (23409–58075)	0.022
Total CD36+ MVs	2161 (1274–3213)	2754 (1704–3772)	NS
EMVs	157 (119–238)	282 (204–522)	0.002
CD36+ EMVs	143 (85–212)	212 (170–408)	0.008
PMVs	2020 (1387–2876)	1664 (1382–2773)	NS
CD36+ PMVs	1758 (1229–2475)	1482 (1219–2482)	NS
MMVs	5 (3–11)	10 (7–14)	0.004
CD36+ MMVs	3 (2–4)	5 (4–8)	0.001
ErytMVs	207 (147–282)	296 (193–453)	0.043
CD36+ ErytMVs	8 (3–13)	11 (6–15)	NS
TF+ MPs	225 (204–396)	408 (327–451)	0.006

Values (MVs *μ*L^−1^ plasma) are shown as the median (interquartile range). MVs, microvesicles; MMVs, monocyte-derived MVs; EMVs, endothelial cell-derived MVs; PMVs, platelet-derived MVs; ErytMVs, erythrocyte-derived MVs; CD36, scavenger receptor CD36; TF, tissue factor.

**Table 3:** Multiple regression analysis with oxLDL-C and gender as independent variables.

	Controls		FH ATX−		FH ATX+	
	*β*	*p* value	*β*	*p* value	*β*	*p* value
	*R* ^2^ = 0.05		*R* ^2^ = 0.03		*R* ^2^ = 0.76	

Total CD36+ MVs						
oxLDL-C	−0.07	NS	0.16	NS	0.94	<0.001
Gender	0.18	NS	−0.08	NS	0.53	0.004

	*R* ^2^ = 0.01		*R* ^2^ = 0.03		*R* ^2^ = 0.64	

CD36+ PMVs						
oxLDL-C	−0.08	NS	0.11	NS	0.88	<0.001
Gender	0.04	NS	−0.13	NS	0.32	NS

	*R* ^2^ = 0.09		*R* ^2^ = 0.16		*R* ^2^ = 0.41	

CD36+ MMVs						
oxLDL-C	0.06	NS	0.30	NS	0.58	0.027
Gender	0.33	NS	0.24	NS	0.60	0.024

	*R* ^2^ = 0.08		*R* ^2^ = 0.01		*R* ^2^ = 0.68	

CD36+ EMVs						
oxLDL-C	−0.20	NS	0.02	NS	0.90	<0.001
Gender	0.14	NS	−0.07	NS	0.39	0.041

	*R* ^2^ = 0.04		*R* ^2^ = 0.16		*R* ^2^ = 0.51	

CD36+ ErytMVs						
oxLDL-C	−0.19	NS	0.26	NS	0.78	0.003
Gender	0.03	NS	0.28	NS	0.27	NS

The standardized regression coefficients beta (*β*) is included as a measure of how strongly each predictor variable influences the criterion (dependent) variable. *p* values are two-sided and considered significant when <0.05. See Table 2 for abbreviations.

## Abbreviations

ATX: Achilles tendon xanthomas
CD36: Cluster of differentiation 36
EMVs: Endothelial cell-derived MVs
ErytMVs: Erythrocyte-derived MVs
FH: Familial hypercholesterolemia
FSC-A: Forward scatter area
MVs: Microvesicles
MMVs: Monocyte-derived MVs
oxLDL-C: Oxidized LDL-cholesterol
PFP: Platelet-free plasma
PMVs: Platelet-derived MVs
PS: Phosphatidylserine
SSC-A: Side scatter area
TF: Tissue factor.
